# Secretaryships

**Published:** 1902-03

**Authors:** 


### SECRETARYSHIPS.

WE want to say a word in favor of these important personages, whose work is not often fully appreciated. Wheels are good things; they go around. Well, it is just this work that these hard-working officials do. They make the wheels go around, they regulate their pace, steadying a too fast one here and quickening a too slow one there. They furnish the oil that keeps them running smoothly, they lessen the friction that might burn out the bearings, and this all counts for much in our true progress. The JOURNAL does not believe that any land in the world can produce as much good secretaryship timber as the United States. Our American Association has been thrice blessed with good officials in this post of responsibility. Many of our State organizations have equally efficient ones at their back, and some of our local societies' whole existence rests on the faithful work of these painstaking, earnest officials. While it is a post of honor, it is one of great responsibility, and there is no limit to the work to be done, and sometimes they are more than ill-paid, even in the gratitude that all can express, and too often some more substantial encouragement might be given that would add to the comfort and pleasure of many of these officials. Do not forget your secretary?

---

ASSOCIATION progress over our land is bearing the richest returns for our profession. State bodies are holding meetings of unusual interest and importance. The programmes have been rich in good things, and every veterinarian should be encouraged and stimulated to greater efforts to discharge his duty in contributing something to this advancement. Minnesota, Iowa, Pennsylvania, and New Jersey have all held unusually interesting meetings since the incoming of the new year.

